# *Yersinia enterocolitica* biotype 1B case report: an unusual pathogen in an osteoarticular infection on device

**DOI:** 10.1186/s12879-020-05204-2

**Published:** 2020-07-11

**Authors:** Frédéric Wallet, Anne-Sophie Le Guern, Malo Penven, Eric Senneville, Cyril Savin, Caroline Loïez

**Affiliations:** 1 CHU Lille, F 59000 Lille, France; 2 Laboratoire de Bactériologie - Institut de Microbiologie, Centre de Biologie Pathologie, F-59037 Lille Cedex, France; 3 Unité de Recherche Yersinia / Centre National de Référence de la peste et autres yersinioses, Institut Pasteur, F75015 Paris, France; 4 CH Tourcoing, F 59200 Tourcoing, France; 5 Univ. Lille, F59000 Lille, France; 6 Centre de Référence des Infections Ostéo-Articulaires Complexes Nord-Ouest (CRIOAC-NO) Lille, Tourcoing, France

**Keywords:** *Yersinia enterocolitica*, Prosthetic device, Osteoarticular infection, Biotype 1B

## Abstract

**Background:**

*Yersinia enterocolitica* is an aero-anaerobic Gram-negative coccobacilli of the *Enterobacteriaceae* family, rarely reported in osteoarticular infection.

**Case presentation:**

This report case described a rare septic osteoarticular infection on device due to *Yersinia enterocolitica* biotype 1B. A purulent fistula appeared after osteosynthesis with plate performed abroad 27 days prior to the presentation for a distal femoral fracture. The treatment consisted of surgical irrigation and washing of the femoral plate and a bitherapy by levoflaxacine and ceftriaxone during 3 months.

**Conclusion:**

*Y. enterocolitica* biotype 1B is extremely rare in France. Moreover, the strain implicated in this european case is extremely close from the USA reference strain (with only 2 SNP difference) described in a septicemia in Ohio. The extreme proximity of the strains underlines the need for a sustained surveillance of the spread of this pathogen in France.

## Background

*Yersinia enterocolitica* is an aero-anaerobic Gram-negative coccobacilli of the *Enterobacteriaceae* family. They can be isolated in the gastrointestinal tract of human, in the animals and in the environment. Based on biochemical reactions, *Y.enterocolitica* strains are subdivided into 6 biotypes: biotype 1A is non-pathogenic, biotypes 2 to 5 are low-pathogenic whereas biotype 1B is highly pathogenic [1, 2]. In human while non-pathogenic biotype 1A strains can be in transit in the gut without causing any symptoms, pathogenic strains cause mainly gastroenteritis with diarrhea, abdominal pain and fever, but also mesenteric lymphadenitis, deep abscess and rarely systemic forms as septicemia. Few cases of osteomyelitis due to *Y.enterocolitica* have been reported but we describe, here, a case of osteoarticular infection (OAI) on a device in an immunocompromised patient.

## Case presentation

A 87-year-old woman was admitted to the traumatology septic ward of the Centre Hospitalier Universitaire of Lille for purulent scar issue after osteosynthesis with plate performed abroad 27 days prior to the presentation for a distal femoral fracture.

On admission, a purulent fistula was observed at the femoral scar. The patient was 37 °C and was hemodynamically stable. Laboratory data included leucocytosis count of 14.80 × 10^9^/l, with 83.7% of polymorphonuclear leukocytes, and C-reactive protein level of 108 mg/l (*N* < 6 mg/l). Surgical treatment by irrigation and washing of the femoral plate was performed. Three bacteriological samples (femoral bone, soft parts and liquid collection) were sent to the laboratory. Direct Gram smear examination of the three samples did not show any bacteria but many polymorphonuclear leukocytes were observed. These samples were plated on polyvitex agar and blood agar before being incubated in aerobic atmosphere at 37 °C, and aerobic and anaerobic blood culture bottles were inoculated before being incubated in BacT/ALERT® VIRTUO™ (bioMérieux, Marcy l’Etoile, France). After 1 day of incubation, cultures were positive for the three samples, yielding bipolar Gram-negative rods. This non-motile, non-spore forming rod was oxidase negative, and produced indole. The identification was performed by Microflex mass spectrometer (Bruker Daltonik, Wissembourg, France). The result of the pattern-matching process was expressed with a score of 2.2 giving *Y.enterocolitica* as first choice. The rapid esculin hydrolysis test performed on the strain was negative, as most strains belonging to virulent serogroups [1].

In vitro antimicrobial susceptibility testing with AST-N233 and AST-XN05 Vitek 2 cards (bioMérieux) was performed as recommended by the CA-SFM 2018 criteria (www.sfm-microbiologie.org). This strain has a wild phenotype with only resistance to amoxicillin, amoxicillin-clavulanate, ticarcillin. A bitherapy by levofloxacine (500 mg per day) and ceftriaxone (1 g per day) was administrated intravenously for 10 days followed by oral levofloxacine (500 mg per day) during 3 months.

The Yersinia National Reference Laboratory (YNRL) confirmed the *Yersinia enterocolitica* species with complete characterization and determination of its pathogenic potential using metabolic tests: API20E and API50CH strips (bioMérieux), tween-esterase and pyrazinamidase activities and seroagglutination. According to the biotyping scheme of Wauters [2], the strain belonged to the biotype 1B. Serotype O:8 was determined and the strain was recorded as IP41365 in the YNRL strain collection. The whole genome of the strain was sequenced with the Nextera XT protocol using a NextSeq 500 Sequencer (Illumina). A de novo assembly of the genome was performed as described by Savin et al. [3]. Sequencing raw data of IP41365 strain have been deposited at NCBI under BioProject PRJNA521039. In silico genomic analysis confirmed the presence of the *Yersinia* high pathogenicity island.

**Fig. 1:**
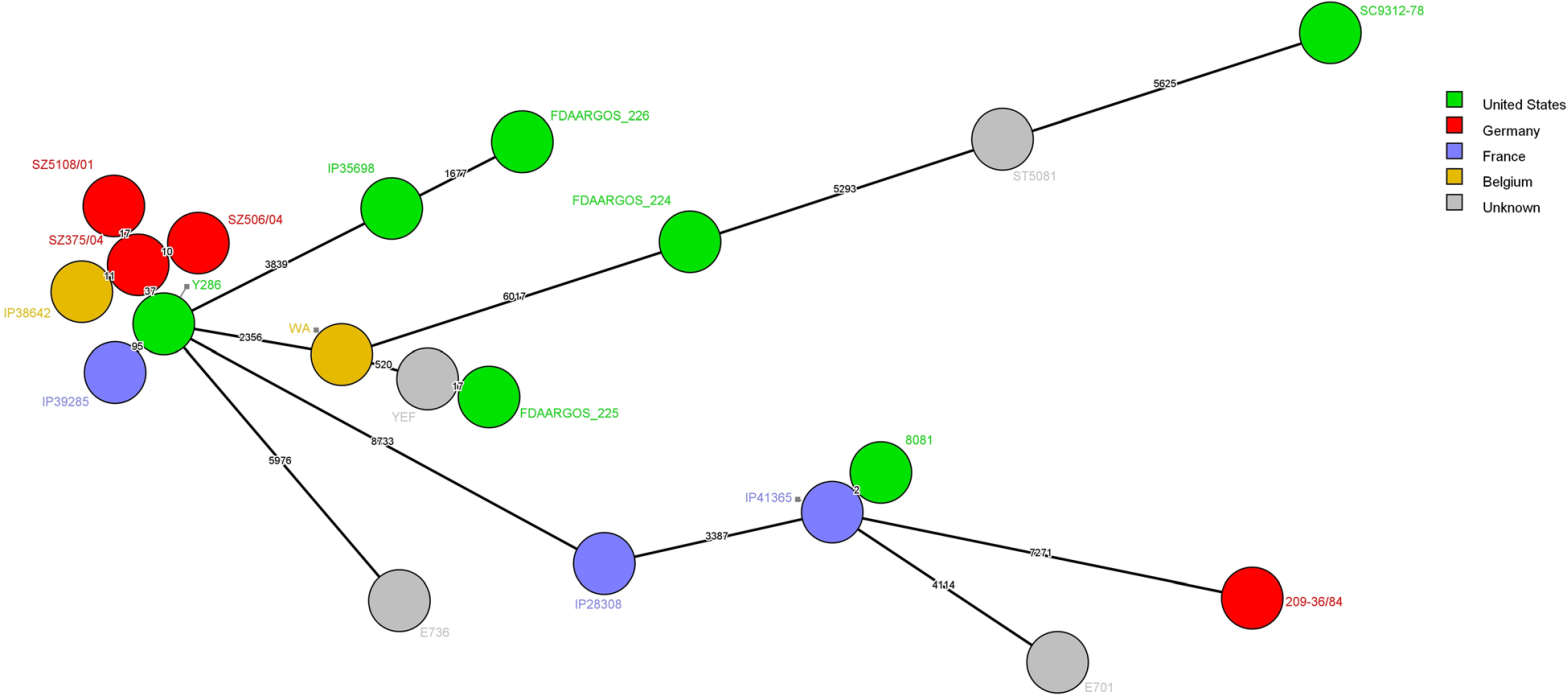
Minimal spanning tree of 20 *Y. enterocolitica* biotype 1B strains compared with wgSNP analysis (Bionumerics 7.6, Applied Maths, Sint-Martens-Latem, Belgium) using 8081 strain as reference (accession number AM286415). Numbers on the branches indicate the difference in SNPs between two strains. Logarithmic scaling was used for branch lengths

Genetic relatedness of IP41365 strain with other sequenced and publicly available genomes of biotype 1B strains was determined as described by Savin et al. [3] (Fig. 1). We observed that closest relative of IP41365 strain is 8081 strain, which is the first *Y.enterocolitica* strain whose genome was sequenced in 2006 [4]. Distance between those strains relies on only 2 SNPs difference. Investigation on acquisition or loss of DNA did not reveal any difference between IP41365 and 8081 strains. Those results mean that the 2 strains are almost the same.

## Discussion and conclusions

Few cases of septic OAI due to *Y.enterocolitica* such as osteomyelitis or spondylodiscitis [5] have been reported. OAI on device are very rare, and only eight cases with our case have been described in literature (Table 1). Most often, the patients are old (> 70 years-old). In all cases, the patients were febrile in the 48 h before the admisssion, except in our case. The OAI due to *Y.enterocolitica* can occur most often in immunocompromised patients such as alcoholism, liver diseases, colic neoplasia, splenectomy, diabetes or iron overload. These characteristics were found in 3/8 cases. In our case, none of these underlying conditions were noted but the patient was treated by hydroxyurea for an essential thrombocythemia. Endly, the presence of foreign device is known to be a predisposing factor for bacterial implantation by the biofilm constitution. The etiological diagnosis was performed by bacteriological analysis of local samples in 8/8 cases whereas blood cultures were positive in only one case. Although our patient did not report any symptoms of septicemia or diarrhea, the gastrointestinal origin of *Y.enterocolitica* led to a digestive exploration showing a diverticulitis: so, this injury may be the source of an asymptomatic bacteriemia with possible dissemination and secondary localisations as osteitis. In the other 7 cases of the literature, diarrheal symptoms were described once (case 5). However, gastrointestinal tract is probably the source of penetration of *Y.enterocolitica*. In case of extra-digestive infection with *Y.enterocolitica*, gastrointestinal investigations are needed to diagnose a potential disease as a diverticulitis or a neoplasia, increasing the risk of translocation as in our case and in case 6.

**Table 1:** Osteoarticular infections on device by *Y. enterocolitica* in the literature

Case	Age / Sex	Material	Underlying disease	Symptoms	Positive sample (biotype, serotype)	Ref
1	83 / F	Hip prothesis	None underlying diseases	No gastrointestinal symptoms Febrile (T = 38.5 °C) at admission	Bone (biotype 2, serotype O:9)	[6]
2	72 / M	Hip prothesis	None underlying diseases	No gastrointestinal symptoms Febrile (T = 39.4 °C) at admission	Bone (biotype 4, serotype O:3)	[7]
3	84 / F	Knee prothesis	None underlying diseases	No gastrointestinal symptoms Febrile at admission	Joint fluid (−, serotype O:9)	[8]
4	14 / F	Vertebral material	None underlying diseases	No gastrointestinal symptoms Febrile (T = 38.2 °C) at admission	Spinal tissue (biotype3, serotype O:5,27)	[5]
5	80 / F	Knee prothesis	Recurrent hemarthrosis Diabetes Iron supplement	Recent diarrhea Febrile (T = 39 °C) at admission	Joint fluid (biotype 4, serotype O:3)	[9]
6	77 / M	Knee prothesis	Hepatocellular carcinoma Diverticulitis	No gastrointestinal symptoms Febrile (T = 38.7 °C) at admission	Joint fluid + Blood culture (biotype and serotype unknown)	[10]
7	90 / M	Knee prothesis	None underlying diseases	No gastrointestinal symptoms Febrile (T = 38.6 °C) at admission	Joint fluid (−, serotype O:3)	[11]
8	87 / F	Femoral plate	Thombocytemia treated by hydroxyurea Diverticulitis	No gastrointestinal symptoms Apyretic at admission	Bone (biotype 1B, sérotype O:8)	This study

Enteric yersiniosis in France is mainly caused by *Y.enterocolitica* belonging to biotypes 4 and 2 but very rarely caused by strains belonging to the highly pathogenic biotype 1B [12]. Strains of biotype 1B caused several outbreaks of gastroenteritis in 1976, 1981, 1995 and 2011 in the United States [13, 14] and in 2004 in Japan [15] while only few sporadic cases were reported in Europe [16]. Surprisingly IP41365 strain belongs to the biotype 1B, which is extremely rare in France with only 3 strains isolated in the past 20 years [3]. In a more surprising way, based on a whole genome SNP analysis, closest relative of IP41365 is 8081 strain with only 2 SNP difference (Fig. 1). The 2 nucleotides difference among 4.6 Mb genome [4] and the absence of acquisition or loss of DNA between the 2 strains suggest that the strains are almost the same. Indeed, distances lower than 11 SNP were observed between *Y.enterocolitica* 4/O:3 strains circulating between pigs and human in Côte d’Ivoire [17]. 8081 strain was isolated in the early 1980’s from a patient presenting with septicemia in Ohio (USA) [18]. As it represents the first sequenced genome of a *Y.enterocolitica* strain, 8081 is now a reference strain in many laboratories working on enteric yersiniosis worldwide. The extreme proximity of the 2 strains would mean that the patient was infected by 8081 strain whereas she did not traveled abroad in the previous few years nor worked in a laboratory specialized in the *Yersinia*. The infection of the patient with IP41365 strain, which is extremely close from 8081 strain, is a mystery, as the strain seems to not having evolved in the last 40 years while traveling across the Atlantic Ocean. The extreme proximity of the strains underlines the need for a sustained surveillance of the spread of this pathogen in France.

## Data Availability

Not applicable to data. All information utilized could be found using the references provided in body of the manuscript.

## Abbreviations

SNP: Single-Nucleotide Polymorphism
OIA: Osteoarticular Infection
N: Normale
CA-SFM: Comité de l’Antibiogramme de la Société Française de Microbiologie
YNRL: Yersinia National Reference Laboratory
DNA: Desoxyribose Nucleic Acid
Mb: Megabase
